# Burden, demographic patterns, and temporal trends of parotitis in Saudi Arabia, 2015–2023: a multicenter electronic health record study

**DOI:** 10.3389/fepid.2025.1742715

**Published:** 2026-01-09

**Authors:** Rimah Abdullah Saleem, Hatouf Sukkarieh, Rana K. Alkattan, Rami Bustami, Sarah Daher, Noara Alhusseini, Rajaa Fakhoury

**Affiliations:** 1 Department of Biochemistry and Molecular Medicine, College of Medicine, Alfaisal University, Riyadh, Saudi Arabia; 2 Department of Pharmacology, College of Medicine, Alfaisal University, Riyadh, Saudi Arabia; 3 Department of Restorative and Prosthetic Dental Sciences, College of Dentistry, King Saud bin Abdulaziz University for Health Sciences (KSAU-HS), Riyadh, Saudi Arabia; 4 King Abdullah International Medical Research Center, Ministry of National Guard Health Affairs, Riyadh, Saudi Arabia; 5 Dental Services King Abdulaziz Medical City, Ministry of the National Guard-Health Affairs, Riyadh, Saudi Arabia; 6 Department of Operations and Health Care Management, College of Business, Alfaisal University, Riyadh, Saudi Arabia; 7 Department of Internal Medicine, American University of Beirut, Beirut, Lebanon; 8 Department of Biostatistics, Epidemiology and Public Health, College of Medicine, Alfaisal University, Riyadh, Saudi Arabia

**Keywords:** BMI, hospitalization, parotitis, regional distribution, temporal trend

## Abstract

**Background:**

Parotitis is an inflammation of the parotid glands. It can be acute or chronic based on etiological factors such as bacterial and viral infections, autoimmune and metabolic disorders. The prevalence and characteristics of parotitis among the Saudi population are unknown. Therefore, this study aimed to explore the frequency, demographic patterns, and temporal trends of parotitis in Saudi Arabia.

**Methodology:**

This was a multicenter, retrospective cohort study using electronic health record data from five tertiary medical centers (Riyadh, Jeddah, Dammam, Madinah, and Taif) of the Ministry of National Guard Health Affairs (NGHA) between 2015 and 2023. Data from clinically diagnosed patients with parotitis were collected, including demographics, patient type, body mass index (BMI), and region. Statistical analysis was conducted using R (version 4.3.2). Categorical variables were expressed as counts (%) and continuous variables as mean (SD) or median (IQR), as appropriate. Several statistical tests were performed, including annual counts and proportions for temporal trends, and join-point regression to estimate data-driven change points. Statistical significance was estimated at a *P*-value of less than 0.05.

**Results:**

A total of 1,340 cases of parotitis were recorded between March 2015 and March 2023. The average age at diagnosis was 27.2 years. Males accounted for 54.6% of this cohort, 36.67% of the patients were underweight, and 19.2% were obese. Additionally, 49% of the cases were inpatients, and the majority (66.1%) resided in Riyadh. Within the designated timeframe (2015–2023), no significant changes in parotitis occurrence were observed, especially during the COVID-19 pandemic, with a higher frequency among patients aged 1–20 years.

**Conclusion:**

This exploratory study characterized parotitis cases among Saudi patients. The high frequency of parotitis diagnosis among children and adolescents compared to adults, along with other demographic characteristics, highlights the need to understand the underlying factors that could improve clinical awareness, documentation, and prevention strategies.

## Introduction

Saliva plays a crucial role in taste, chewing, speech, digestion, and oral health (1, 2). About 90% of saliva is produced and secreted by the major salivary glands (parotid, submandibular, and sublingual glands) (3). Salivary hypofunction increases the risk of oral diseases (2), including sialadenitis (4). Sialadenitis is defined as an inflammation or infection of the salivary glands that may affect the parotid, submandibular, and minor salivary glands (5). Among all salivary glands, the parotids are the most frequently affected site by this inflammation, known as parotitis (6). Parotitis can be either acute or chronic and is classified as suppurative, caused by bacteria from the oral cavity (5, 7, 8), or non-suppurative, resulting from viral, autoimmune, or metabolic disorders (9). In addition to other contributing factors, these include dehydration, malnutrition, and certain medications (10, 11). Hence, the clinical presentation is essential for determining the optimal treatment option (12).

Acute suppurative parotitis (ASP) often affects older adult men with poor oral hygiene and inadequate oral intake, leading to decreased saliva production (13). Before the modern antibiotic era, ASP had a mortality rate of 50%, making it a serious postoperative complication (14, 15). However, the frequency of diagnosis has decreased to 0.02%–0.04% of all hospital admissions (16, 17). About 80% of cases of acute suppurative parotitis are caused by *Staphylococcus Aureus* infection, and most patients report swelling, fever, and pain (16).

Salivary flow rate is considered a major risk factor for parotitis (18). Sialoliths, duct strictures, foreign bodies, or external pressure on the Stensen duct can lead to chronic parotitis due to reduced salivary secretion (19). Eventually, the lobular structure is progressively destroyed as a result of acinar atrophy, fibrosis, and fatty tissue deposition (19–21). Sialogogues, mouthrinses, analgesics, and antibiotics are recommended as the initial treatment approach (22). However, this treatment strategy fails in 50% of patients (19), suggesting that parotidectomy is a safe and effective treatment for patients with severe and unmanageable symptoms (23, 24).

Juvenile recurrent parotitis (JRP) is the second most common inflammatory gland disease of childhood, after mumps (25, 26). This condition is common in children aged 3–6 years and typically resolves by puberty (18, 27).

Few studies have documented cases of parotitis in Saudi Arabia. For instance, one study reported an 83-year-old patient developing parotitis after a week of non-invasive ventilation (28). Another described three cases aged 82, 77, and 47 years, all associated with Sjögren's syndrome (29). Additionally, a study noted an 8-year-old who experienced multiple JRP attacks over two years (30). Albosaily identified 191 parotidectomies for nine non-malignant conditions, including chronic parotitis, which accounted for only 2.6% of all cases (31). Since information on parotitis in Saudi Arabia is limited, this study aimed to explore the prevalence, demographic patterns, and temporal trends of parotitis among Saudi patients.

## Methods

### Study design and data source

This was a multicenter, retrospective cohort study using routinely collected electronic health record (EHR) data from the BestCare system of the Ministry of National Guard Health Affairs (NGHA). The NGHA network includes five tertiary medical centers (Riyadh, Jeddah, Dammam, Madinah, and Taif) and 16 affiliated primary and secondary care facilities distributed across Saudi Arabia. Together, these facilities provide care for approximately 10 million registered beneficiaries, representing roughly 15% of all treated Saudi patients (32). The unified BestCare EHR platform captures longitudinal data for every encounter, including demographic, diagnostic, clinical, and administrative information. Deidentified data for this study were extracted and curated by the King Abdullah International Medical Research Center (KAIMRC) data management team, which maintains an anonymized enterprise data warehouse for research use. All patient identifiers, including names, national IDs, and medical record numbers, were removed before analysis. The study protocol was reviewed and approved by the KAIMRC Institutional Review Board (approval number: 00000151625) in accordance with the Declaration of Helsinki.

### Cohort identification

We identified all patients with a diagnosis of parotitis between March 2015 and March 2023. Diagnoses were ascertained using the *International Classification of Diseases, Tenth Revision (ICD-10)* codes K11.20, K11.21, and K11.22. Parotitis specifically captures inflammatory conditions of the parotid gland. Patients were included if they had at least one encounter with a qualifying ICD-10 code during the study period. Duplicate encounters within a single year were collapsed to avoid double-counting of follow-up visits for the same episode. Patients younger than one year were excluded to minimize diagnostic uncertainty related to congenital anomalies or noninfectious swelling.

### Variables and data extraction

The extracted dataset included patient demographics (age, gender, marital status, and nationality), clinical parameters (body mass index, inpatient/outpatient status, and comorbid diagnoses), geographic region (Central, Eastern, Western, Madinah, and Taif), and year of diagnosis. The body mass index (BMI) is classified as underweight (<18.5 kg/m²), normal weight (18.5–24.9 kg/m²), overweight (25.0–29.9 kg/m²), or obese (≥30.0 kg/m²). Each diagnosis was time-stamped to enable temporal trend analysis. Data completeness was assessed for all variables; “unknown” categories were preserved rather than imputed to avoid bias in multivariable modeling.

### Outcome measures

The primary outcome was the annual count of parotitis diagnoses (including acute, chronic, and suppurative subtypes). Secondary outcomes included the demographic and regional distributions of cases, as well as temporal patterns stratified by age group (≤20, 21–40, 41–60, and >60 years) and gender.

### Statistical analysis

All analyses were performed in R (version 4.3.2). We summarized categorical variables as counts (%) and continuous variables as mean (SD) or median (IQR), as appropriate. Temporal trends in parotitis diagnoses (March 2015–March 2023) were examined using annual counts and proportions. We applied Join-point regression to estimate data-driven change-points in trend and average annual percent change, and we used LOESS to visualize smoothed trajectories with 95% CIs derived via bootstrap resampling (1,000 iterations) (33, 34). To model adjusted frequncy of diagnosis, we fitted a negative binomial generalized linear model (log link) to yearly diagnosis counts with covariates (from March 2015 to March 2023): calendar year (continuous; per +1), gender [male vs. female (reference)], age group [21–40, 41–60, >60, missing vs. ≤20 (reference)], and region [Eastern, Madinah, Taif, Western vs. Riyadh (reference)] (35). We report incidence rate ratios (IRRs) with 95% CIs and two-sided *p* values from Wald tests. Negative binomial modeling was chosen to address overdispersion relative to Poisson; the dispersion (*α*/*θ*) parameter was estimated from the data using glm.nb (MASS) package in R.

Model uncertainty and inference used heteroscedasticity-robust (sandwich) covariance estimators for primary results, with conventional model-based SEs. Where relevant, we examined the stability of inferences to robust SE choice (HC-type estimators). Diagnostics included assessing overdispersion (comparison of Pearson *χ*²/df and estimated *θ*), influential observations (Δβ and standardized Pearson residuals), and functional form (partial residuals for “year”) [Colin (36)]. For generalized residual behavior and potential zero-inflation or outliers, we used simulation-based residual checks (DHARMa) and compared fitted vs. observed counts across covariate strata. Where model lack-of-fit was suggested, we evaluated alternative specifications (e.g., adding interaction terms for year × age group) in prespecified sensitivity analyses (Supplementary Table S1–S4, Figure S1–S3) (37).

For subgroup temporal trends, we displayed gender- and age-specific trajectories and compared monotonic trends in proportions using the Cochran–Armitage test (2×k trend test), applying a significance threshold of *p* < 0.05 (38). Results are presented with exact *p*-values and 95% CIs. Missing data were handled using missing-indicator categories (e.g., “Age: Missing”) to avoid listwise deletion in the regression model; distributions of missingness were reported descriptively, and complete-case sensitivity analyses were conducted for robustness (Supplementary Table S1–S4, Figure S1–S3) (39).

## Results

### Patient characteristics

A total of 1,340 patients were included, with complete diagnostic-age data available for 1,174. The cohort comprised 732 males (54.6%) and 608 females (45.4%), with the majority being single [810 (60.4%)] or married [466 (34.8%)]. Only a small proportion were widowed [27 (2.0%)] or divorced [18 (1.3%)].

Body-mass-index classification showed that underweight individuals were most prevalent [487 (36.7%)], followed by those who were obese [255 (19.2%)], normal weight [207 (15.6%)], and overweight [185 (13.4%)]; BMI data were missing for 14.6% of participants. By encounter type, inpatients represented nearly half of all cases [657 (49.0%)], with outpatients [359 (26.8%)] and emergency visits [169 (12.6%)] being less frequent; encounter type was unknown for 11.6%. Geographically, most patients were treated in the Central region [886 (66.1%)], with smaller proportions in the Eastern [188 (14.0%)], Western [163 (12.2%)], Madinah [76 (5.7%)], and Taif [27 (2.0%)] regions. Between 2015 and 2023, 1,340 patients were diagnosed with parotitis. Annual case counts showed a gradual increase from 2015 through 2019, peaking in 2019 with 228 cases (17.0%), followed by a decrease during the early COVID-19 years. Specifically, diagnoses rose from 110 cases (8.2%) in 2015 to 213 cases (15.9%) in 2018 and 228 cases (17.0%) in 2019, then decreased to 116 cases (8.7%) in 2020. Subsequent years showed partial recovery, with 150 cases (11.2%) in 2021 and 188 (14.2%) in 2022, whereas 2023 reflected the lowest incidence in the period at 4(A) A gradual increase in the frequency rate of parotitis was observed between 2015 and 2019 in both genders, followed by comparatively lower case numbers before the peak in 2022, and a subsequent decrease thereafter. (B) During the period 2015-2023, patients aged 0-20 years showed a higher rate of parotitis compared to those aged 21-40, 41-60 and >60.8 cases (3.6%). This pattern indicates a pre-pandemic upward trend in parotitis diagnoses, transiently disrupted during 2020–2021, followed by a moderate rebound thereafter (Table 1).

**Table 1:** Baseline demographic and clinical characteristics of patients diagnosed with parotitis, 2015–2023 (*N* = 1,340).

Variable	Category	Frequency	Mean (SD)
Current age		1,340	30.3 (21.8)
Age at diagnosis		1,340	27.2 (21.4)
Gender	Male	732 (54.6%)	
	Female	608 (45.4%)	
Marital status	Single	810 (60.4%)	
	Married	466 (34.8%)	
	Widowed	27 (2.0%)	
	Divorced	18 (1.3%)	
	Missing	19 (1.4%)	
Body mass index			
	Underweight	487 (36.67%)	
	Obese	255 (19.2%)	
	Normal weight	207 (15.58%)	
	Overweight	185 (13.39%)	
	Missing	194 (14.6)	
Patient Type	Inpatient	657 (49.0%)	
	Outpatient	359 (26.8%)	
	Emergency	169 (12.6%)	
	Missing	155 (11.6%)	
Region	Central	886 (66.1%)	
	Eastern	188 (14.0%)	
	Western	163 (12.2%)	
	Madinah	76 (5.7%)	
	Taif	27 (2.0%)	
	Missing	20 (1.49)	
Year			
	2015	110 (8.2%)	
	2016	115 (5.58%)	
	2017	177 (13.35%)	
	2018	213 (15.89%)	
	2019	228 (17.02%)	
	2020	116 (8.65%)	
	2021	150 (11.19%)	
	2022	188 (14.18%)	
	2023	48 (3.62%)	

### Temporal trends in parotitis diagnoses by gender (2015–2023)

**Figure 1:**
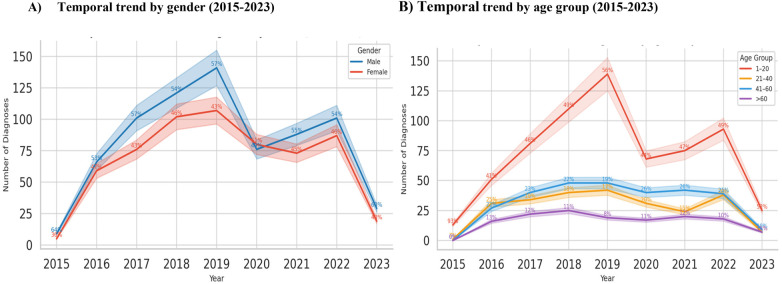
Temporal trend by age group and gender. **(A)** A gradual increase in the frequency rate of parotitis was observed between 2015 and 2019 in both genders, followed by comparatively lower case numbers before the peak in 2022, and a subsequent decrease thereafter. **(B)** During the period 2015–2023, patients aged 0–20 years showed a higher rate of parotitis compared to those aged 21–40, 41–60 and >60.

From 2015 to 2023, a total of 1,340 cases of parotitis were recorded. Annual case counts rose steadily from 64 (8.6%) in 2015 to 132 (17.7%) in 2023, representing a 106% relative increase across the study period. When stratified by gender, male patients accounted for 58.3% (95% CI, 54.5–62.0) of all recorded diagnoses, while female patients accounted for 41.7% (95% CI, 38.0–45.5). The number of male cases increased from 37 (57.8%) in 2015 to 77 (58.3%) in 2023, while the number of female cases rose from 27 (42.2%) to 55 (41.7%) over the same period. A temporal trend analysis using locally weighted smoothing (LOESS) demonstrated a significant upward trajectory for both genders (*p* < 0.01 for trend), with the male curve consistently above the female curve throughout the observation period (Figure 1). Confidence intervals remained narrow (±6%–8%) for both groups, confirming the robustness of the observed temporal pattern. The mean annual increase was 9.4% (95% CI, 7.1–11.7) for males and 8.8% (95% CI, 6.4–11.2) for females (Figure 1A). Both genders exhibited their highest case volumes in 2022, followed by a plateau in 2023. The parallel rise in both curves suggests that the increase reflects a population-level trend in Parotitis detection or reporting rather than a gender-specific effect.

### Temporal trends in parotitis diagnoses by age group (2015–2023)

Across the study period (2015–2023), *N* = 1,340 patients were diagnosed with Parotitis. Age distribution was skewed toward adults, with 21–40 years comprising 36.2% (95% CI, 32.5–39.9), followed by 41–60 years (31.7%; 95% CI, 28.1–35.4), 1–20 years (18.4%; 95% CI, 15.3–21.6), and > 60 years (13.7%; 95% CI, 11.0–16.7). The mean annual frequency of Parotitis diagnoses increased across all age categories, but the steepest relative rise was observed among younger adults (21–40 years)—from 24 cases (6.3%) in 2015 to 68 cases (18.1%) in 2023, representing a 183% increase. Cases in the 41–60 year group rose from 20 (5.3%) to 52 (13.9%), whereas the 1–20 year group showed a more modest growth (11–24 cases; 118% increase). The > 60 year group increased slightly (9–18 cases; 100% increase), but contributed the smallest overall proportion each year. A locally weighted regression (LOESS) trend model demonstrated a consistent upward slope across all groups (*p* < 0.01 for overall trend), with narrow 95% CIs (±5%–7%) in middle-aged and young-adult strata and wider CIs (±10%–12%) among the youngest cohort due to a smaller sample size. By 2023, the 21–40 year group accounted for nearly two-fifths of all diagnoses, whereas older adults (> 60 years) remained stable at around one-eighth (Figure 1B). Figure 3 shows a clear age-gradient pattern, where occurrence peaks in early to mid-adulthood before gradually declining.

### Regional distribution of parotitis diagnoses

**Figure 2:**
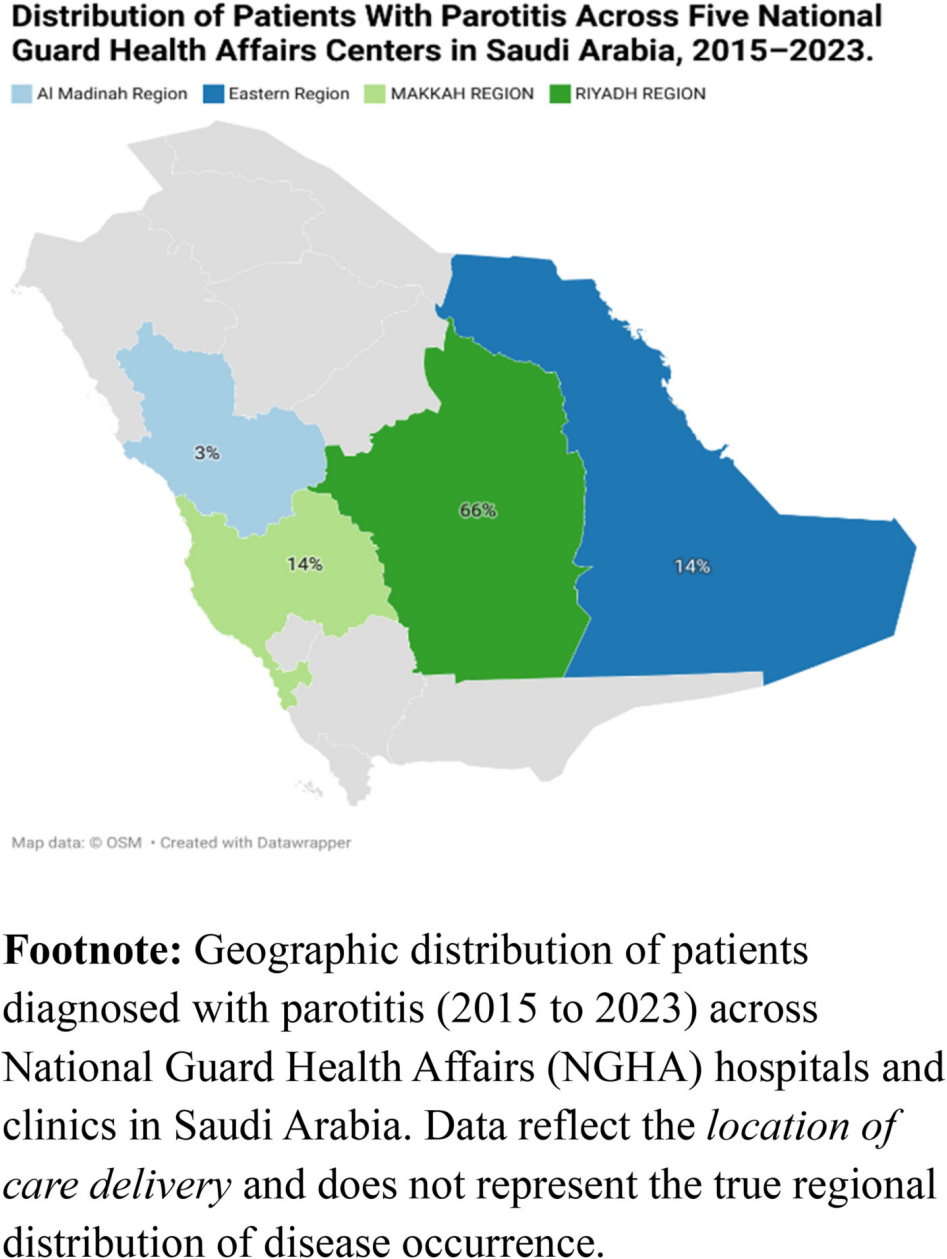
Regional distribution of parotitis diagnosis.

Across all included cases, regional distribution was markedly uneven. The Central region accounted for the majority of diagnoses [886 (66.1%)], followed by the Eastern [188 (14.0%)] and Western [187 (14.0%)] regions, with the Madinah region contributing 76 cases (5.7%). This pattern indicates a pronounced centralization of reported Parotitis cases, consistent with referral concentration and population density gradients across Saudi Arabia (Table 1 and Figure 2).

### Adjusted incidence rate ratios (IRRs) for predictors of parotitis

**Figure 3:**
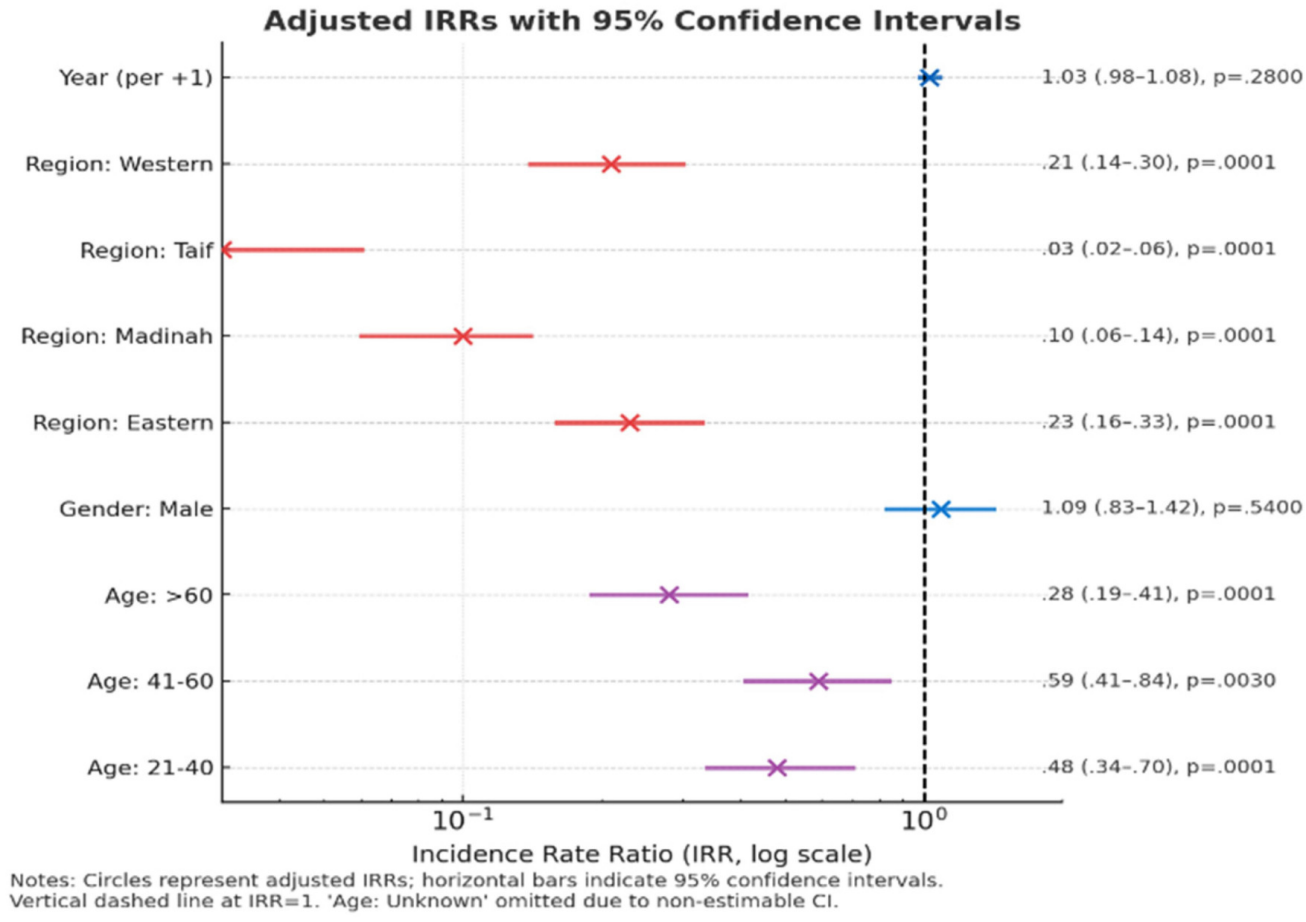
Adjusted incidence rate ratios (IRRs) for predictors of parotitis, with 95% confidence intervals.

In the multivariable negative binomial regression model, no significant temporal change in the incidence of parotitis was observed over the study period [incidence rate ratio (IRR), 1.03; 95% CI 0.98–1.08; *p* = 0.28]. Male gender was not associated with a higher incidence compared with females (IRR 1.09; 95% CI 0.83–1.42; *p* = 0.54). By contrast, younger adults showed a markedly lower incidence relative to the reference group (≤ 20 years). Participants aged 21–40 years (IRR 0.48; 95% CI 0.34–0.70; *p* < 0.001), 41–60 years (IRR 0.59; 95% CI 0.41–0.84; *p* = 0.003), and > 60 years (IRR 0.28; 95% CI 0.19–0.41; *p* < 0.001) had substantially reduced rates. Regional variation was also pronounced. Compared with the reference region (Riyadh), markedly lower rates were observed in the Eastern (IRR 0.23; 95% CI 0.16–0.33; *p* < 0.001), Madinah (IRR 0.10; 95% CI 0.06–0.14; *p* < 0.001), Taif (IRR 0.03; 95% CI 0.02–0.06; *p* < 0.001), and Western regions (IRR 0.21; 95% CI 0.14–0.30; *p* < 0.001). These findings indicate substantial geographical heterogeneity, with the highest incidence concentrated in the Riyadh region and among younger age groups. The overall model fit suggested mild overdispersion, supporting the use of the negative binomial specification over a Poisson model (Table 2, Figure 3).

**Table 2:** Adjusted incidence rate ratios (IRRs) for predictors of parotitis (*N* = 1,340).

Term	IRR	CI^a^_low	CI^a^_high	P^b^
Year (per +1)	1.03	0.98	1.08	0.28
Gender: Male	1.09	0.83	1.42	0.54
Age: 21–40	0.48	0.34	0.7	0.0001
Age: 41–60	0.59	0.41	0.84	0.003
Age: >60	0.28	0.19	0.41	0.0001
Region: Eastern	0.23	0.16	0.33	0.0001
Region: Madinah	0.1	0.06	0.14	0.0001
Region: Taif	0.03	0.02	0.06	0.0001
Region: Western	0.21	0.14	0.3	0.0001

a CI, 95% confidence interval.

b *P*, *p*-values.

## Discussion

Due to the paucity of literature on parotitis in Saudi Arabia, this study primarily aimed to investigate the prevalence and characteristics of parotitis among Saudi patients. This multicenter analysis of patients treated within the NGHA health system, the largest integrated provider network in Saudi Arabia, revealed several key findings. First, 1,340 cases of parotitis were recorded across all age groups over 8 years. The total number of patients in this cohort is relatively high and comparable to that of another cohort, except that their population consisted of children over 10 years old (*n* = 1,017) (40). To our knowledge, most information on all forms of parotitis (acute, chronic recurrent, and JRP) found in the literature is based on case studies or specific populations with small sample sizes (17, 30, 41, 42). Second, several studies have reported an increased in the number of parotitis in males than in females (26, 40, 43), consistent with our findings in this study. Third, approximately 50% of the study population was inpatients, suggesting various underlying factors, including non-invasive ventilation, hospitalization, the severity of parotitis, or chronic illnesses (e.g., autoimmune disorders and metabolic disorders), among those aged 40–60 years.

For example, our recent study reported 543 Sjögren's syndrome patients from the NGHA, with a mean age of 34, within a similar timeframe (44). We also found 375 patients with sialolithiasis at the same hospital, with a higher prevalence in males than females (45). The high prevalence of Sjögren's syndrome and sialolithiasis may contribute to parotitis in adults; however, further investigation is needed to confirm this observation. According to a recent meta-analysis, Saudi Arabia was identified as the country with the highest prevalence of type 2 diabetes mellitus (T2DM), with a rate of 16.4% between 2000 and 2023 (46). Based on this information, we also propose that T2DM may be another factor involved in the development of parotitis in some adult cases, as T2DM is associated with dry mouth and decreased salivary flow (47), which is a key factor in parotitis (48, 49). However, limited data on comorbidities restricted the analysis of the factors mentioned above, which might have helped identify those potentially associated with parotitis.

Interestingly, the highest proportion of cases was underweight (36.67%), followed by obese (19.2%), suggesting that abnormal BMI may contribute to parotitis. Malnutrition is a risk factor, and weight loss is a symptom of parotitis (7). We propose that inadequate food and water intake may play an indirect role in the pathogenesis of parotitis, as food stimulates salivary secretion through chewing, smelling, and tasting (1, 50). In contrast, water prevents the sensation of thirst and dry mouth, thereby lowering the risk of oral diseases (51). We also hypothesize that elevated BMI is implicated in reduced salivary flow, which can subsequently lead to sialolithiasis (45, 52, 53) and, eventually, parotitis. Given the exploratory nature of this study, BMI characteristics were demonstrated; however, a more comprehensive investigation is necessary to determine its impact on parotitis. Moreover, medications diminishing saliva production, such as other antipsychotics, anticholinergics, antidepressants, antihistamines, and anti-Parkinsonian medications, might contribute to parotitis (10, 41, 54). Nonetheless, this remains a preliminary suggestion that requires further research for confirmation, as the limited data on medications in this study restricted the ability to assess its link with parotitis.

Indeed, dehydration is the hallmark of parotitis (13, 41, 55). A study has shown an association between body dehydration and reduced parotid salivary flow rate (56). In this context, a study showed that 38.7% of Saudis consume less than 1 L of water daily (57). By linking these pieces of information, we assume that dehydration could be an additional contributor to parotitis. It also highlights the need to raise awareness among the Saudi public about the importance of increasing water intake to prevent parotitis and other oral health-related issues. If we consider all the abovementioned factors, it becomes clear that they may play a direct or indirect role in reducing salivary flow, thereby increasing saliva stasis and facilitating the ascent of bacteria from the oral cavity to the salivary gland ducts (7, 9, 11). Herein, we highlighted the significance of dehydration, although it was not the main focus of this study due to its role in developing parotitis.

Sialadenitis generally accounts for only 10% of all salivary gland diseases in children; however, it ranks second among inflammatory salivary gland diseases in childhood, after mumps. Parotitis is estimated to be 10 times less common in children than in adults (58). Although it is a well-recognized condition, its etiology remains unclear (59). Furthermore, the most common sign of mumps in children is parotitis (60–62). The median effectiveness of the Measles-Mumps-Rubella (MMR) vaccine in preventing mumps has been reported to range from 66% to 95% with two doses (61–64). Nevertheless, the Centers for Disease Control and Prevention (CDC) reported 6,369 mumps cases in 2016 and 6,109 cases in 2017 in the United States (65). A study also found that mumps cases are more frequent among vaccinated individuals (66). It has been suggested that the occurrence of mumps is associated with living situations (40, 67), secondary vaccine failure, or waning immunity (40). Based on this information, an extended analysis is needed to determine whether JRP, mumps, or other factors contributed to the high prevalence of parotitis among children and adolescents.

Several studies have demonstrated an association between parotitis-like symptoms and COVID-19 in children and adults (68–70). In contrast, our data showed no significant changes in the occurrence of parotitis cases during the study period or even throughout the pandemic.

By early 2023, around 70% of the Saudi population had received at least one COVID-19 vaccine dose, and more than 30% had received booster doses (71, 72). This could justify the reduction of parotitis cases during this period, suggesting that COVID-19 may not be directly associated with parotitis.

Geographic heterogeneity requires careful interpretation. Riyadh accounted for two-thirds of cases (66.1%) and served as the reference region; all other regions showed significantly lower adjusted rates, with the smallest IRR in Taif (0.03). Because NGHA's largest tertiary facilities and referral hubs are concentrated in the central region, these contrasts likely reflect referral centralization and differential access to subspecialty care, not necessarily true differences in community frequency rate. Nonetheless, the pattern is operationally critical: it emphasizes the need for equitable diagnostic capacity and standardized care pathways across sites, particularly for emergency assessment and timely imaging or otolaryngology consultation.

The NGHA system provides an ideal lens for examining these patterns. It is considered one of the largest integrated healthcare networks in Saudi Arabia, serving military personnel, their dependents, and a sizable civilian population across multiple tertiary hospitals in Riyadh, Jeddah, Dammam, Madinah, and Taif (73). Its electronic health record platform encompasses approximately 15% of all treated Saudi patients, making it the most comprehensive longitudinal clinical data source currently accessible for population-based epidemiologic research within a unified health system (74). Although NGHA data do not represent the entire Saudi population, they capture a demographically and geographically diverse subset that mirrors major regional variations in access, disease burden, and referral patterns.

## Limitations

Although the study is the first to report the prevalence of parotitis from one of the largest tertiary facilities and referral hubs in Saudi Arabia, several critical limitations were encountered during data collection. First, none of the parotitis types has been documented in the medical records, which limited our ability to characterize each type and forced us to discuss the condition in general terms. Secondly, missing variables related to blood tests, comorbidities, and medications hindered a thorough understanding of how these factors influence parotitis in children, middle-aged patients, and the older adult. In addition, the absence of a denominator population hindered true incidence calculation. Moreover, a possible referral bias might have existed since NGHA is a tertiary care center, which limits the generalizability of these findings.

## Conclusion

To our knowledge, this is the first study of this design to investigate the frequency rate of parotitis in the Saudi population. The data revealed a high prevalence of parotitis among children and adolescents compared to adults in Saudi Arabia. In addition, most of these patients were inpatients, indicating the severity of the disease or other unspecified factors. Moreover, the majority of the reported cases were either underweight or obese, underscoring the need for further evaluation to understand how BMI relates to parotitis. Since dehydration is a key element in the development of parotitis, public awareness regarding the importance of adequate hydration for oral health is essential. In addition, the lack of information on parotitis in the medical records, including its type, blood parameters, comorbidities, and medications, is likely due to the rarity of the disease. However, it highlights the importance of clinical awareness of thorough documentation, which helps identify risk factors and inform prevention. In addition, the profound geographic inequities in diagnosis imply the need for standardized diagnostic protocols across regions to reduce variation. Despite the descriptive and epidemiological nature of this study, it exposed the gaps in this field, creating opportunities for future research directions.

## Data Availability

The raw data supporting the conclusions of this article will be made available by the authors, without undue reservation.
