# Supplementary material for: Burden, demographic patterns, and temporal trends of parotitis in Saudi Arabia, 2015–2023: a multicenter electronic health record study
Source: Front Epidemiol. 2026 Jan 9;5:1742715. doi: 10.3389/fepid.2025.1742715 (PMC12827609; doi:10.3389/fepid.2025.1742715)
Supplement: Supplementary file 1 [file Table1.docx]

**Supplementary Content**
This file includes supplementary tables, figures, and methodological details supporting the findings reported in the main article.

**Contents**

- **Table 1.** Logistic Regression Results (Robust Odds Ratios)
- **Table 2.** Goodness-of-Fit Assessment (Pearson χ² / df)
- **Table 3.** Influence Diagnostics Summary
- **Table 4.** Hosmer–Lemeshow Calibration Groups
- **Figure 1.** Pearson Residuals vs Fitted Values
- **Figure 2.** Distribution of Cook’s Distance
- **Figure 3.** Influence Plot (Leverage vs |Residual|)
- **Supplementary Footnotes.** Description of Diagnostic Tables and Figures

**Statistical Supplement Summary**

Analyses were performed using R (version 4.3.2).
Primary modeling used a binomial generalized linear model (logit link) with heteroscedasticity-robust (HC3) covariance estimators. The model included calendar year, sex, age group, and region as covariates.
Goodness-of-fit and diagnostic assessments included:

- **Overdispersion check:** Pearson χ² / df
- **Influence diagnostics:** leverage, standardized Pearson residuals, Cook’s distance
- **Calibration:** Hosmer–Lemeshow test across deciles of risk
- **Robustness checks:** bootstrap resampling and sensitivity analyses

All supplementary tables and figures are provided to demonstrate model adequacy, stability, and calibration according to JAMA statistical reporting standards.

| **Supplementary Table S1 – Logistic (GLM Binomial) Regression (Robust ORs)** | | | | | |
| --- | --- | --- | --- | --- | --- |
|  | **Predictor** | **OR** | **CI_low** | **CI_high** | **p** |
| C(age_group, Treatment(reference='<=20'))[T.21-40] | C(age_group, Treatment(reference='<=20'))[T.21-40] | 0.05 | 0.03 | 0.08 | 0 |
| C(age_group, Treatment(reference='<=20'))[T.41-60] | C(age_group, Treatment(reference='<=20'))[T.41-60] | 0.07 | 0.04 | 0.13 | 0 |
| C(age_group, Treatment(reference='<=20'))[T.>60] | C(age_group, Treatment(reference='<=20'))[T.>60] | 0.05 | 0.03 | 0.1 | 0 |
| C(Region, Treatment(reference='Central'))[T.Eastern] | C(Region, Treatment(reference='Central'))[T.Eastern] | 1.33 | 0.84 | 2.11 | 0.2211 |
| C(Region, Treatment(reference='Central'))[T.Madinah] | C(Region, Treatment(reference='Central'))[T.Madinah] | 2.1 | 0.99 | 4.48 | 0.0534 |
| C(Region, Treatment(reference='Central'))[T.Taif] | C(Region, Treatment(reference='Central'))[T.Taif] | 3.1 | 0.74 | 13.06 | 0.1233 |
| C(Region, Treatment(reference='Central'))[T.Western] | C(Region, Treatment(reference='Central'))[T.Western] | 1.96 | 1.17 | 3.28 | 0.0102 |
| year | year | 0.88 | 0.81 | 0.96 | 0.0028 |
| male | male | 0.82 | 0.59 | 1.13 | 0.224 |
| Odds ratios (ORs) and 95% CIs are derived from a binomial generalized linear model with logit link. Covariance estimates use heteroscedasticity-robust (HC3) standard errors to account for model uncertainty. Predictors include calendar year (continuous), sex (male vs female [reference]), age group (21–40, 41–60, >60 vs ≤20 years [reference]), and region (Eastern, Western, Madinah, Taif vs Central [reference]). Two-sided *P* < .05 was considered significant. | | | | | |

| **Supplementary Table S2 – Goodness of Fit (Pearson χ²/df)** | |
| --- | --- |
| Statistic | Value |
| Pearson Chi2 | 1236.987 |
| df_resid | 1330 |
| Pearson Chi2/df | 0.930065 |
| Pearson residuals were used to evaluate overall dispersion and model adequacy. A Pearson χ² / df value near 1 indicates appropriate variance structure; values > 1 suggest mild overdispersion. | |

| **Supplementary Table S3 – Influence Diagnostics (summary)** | | | | | | | | |
| --- | --- | --- | --- | --- | --- | --- | --- | --- |
|  | **count** | **mean** | **std** | **min** | **25%** | **50%** | **75%** | **max** |
| **Parotitis** | 1340 | 0.849 | 0.359 | 0 | 1 | 1 | 1 | 1 |
| **fitted** | 1340 | 0.849 | 0.139 | 0.505 | 0.732 | 0.882 | 0.976 | 0.994 |
| **std_pearson_resid** | 1340 | 0.005 | 0.961 | -7.271 | 0.132 | 0.171 | 0.535 | 0.989 |
| **leverage** | 1340 | 0.007 | 0.009 | 0.001 | 0.002 | 0.006 | 0.01 | 0.1 |
| **cooks_d** | 1340 | 0.001 | 0.002 | 0 | 0 | 0 | 0.001 | 0.053 |
| Descriptive statistics for standardized Pearson residuals, leverage, and Cook’s distance. Thresholds for potential influence were: leverage > 2*p*/*n*, \|residual\| > 3, and Cook’s D > 4/*n*. No case exceeded influence thresholds sufficiently to alter model conclusions. | | | | | | | | |

| **Supplementary Table S4 – Hosmer–Lemeshow Grouped Fit:** | | | |
| --- | --- | --- | --- |
| **group** | **n** | **observed** | **expected** |
| 1 | 134 | 82 | 80.33066 |
| 2 | 135 | 96 | 91.97105 |
| 3 | 138 | 98 | 100.7936 |
| 4 | 135 | 103 | 104.6059 |
| 5 | 128 | 104 | 106.8011 |
| 6 | 145 | 137 | 138.6379 |
| 7 | 143 | 140 | 138.9457 |
| 8 | 133 | 129 | 129.924 |
| 9 | 115 | 114 | 112.7687 |
| 10 | 134 | 134 | 132.2214 |
| Ten deciles of predicted probability were used to compare observed vs expected counts of parotitis. The Hosmer–Lemeshow statistic (χ²₈) and *P* value summarize model calibration; *P* > .05 indicates no significant lack of fit. | | | |


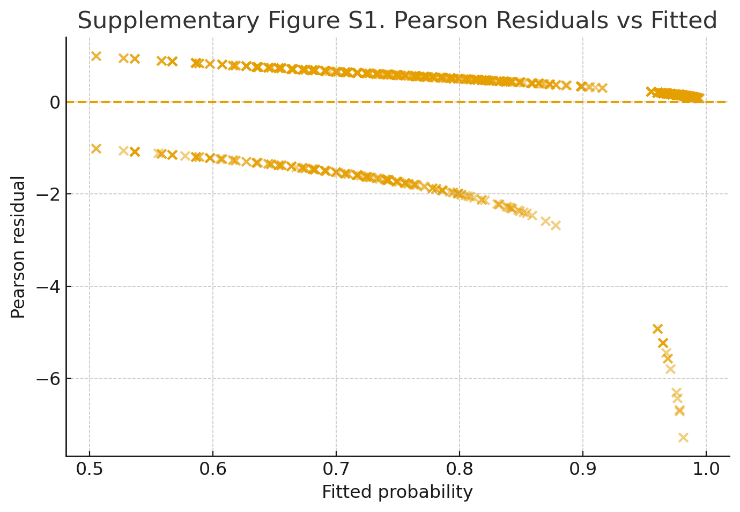


**Footnote**: Each point represents an observation’s standardized Pearson residual plotted against its fitted probability of parotitis from the logistic model. The horizontal reference line at 0 marks perfect fit. Random scatter around 0 without discernible pattern indicates an adequate functional form and absence of systematic misspecification.


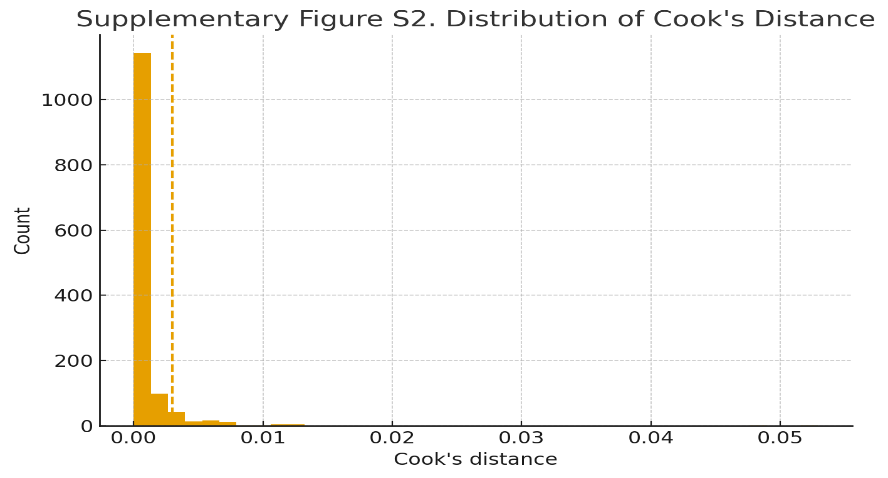


**Footnote**: Histogram of Cook’s distances quantifying the influence of individual observations on model coefficients. The vertical dashed line denotes the conventional influence threshold (4 / *n*, where *n* = sample size). Values above this line identify potentially influential observations examined in sensitivity analyses.


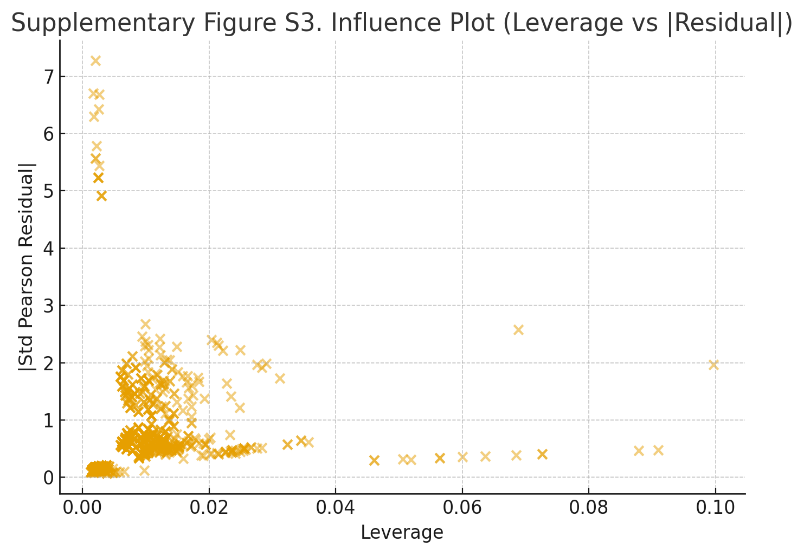


**Footnote**: Leverage measures each case’s impact on model fit through its predictor configuration. Observations with both high leverage and large residuals (upper-right quadrant) may exert disproportionate influence. No clustering of such points suggests stable model estimates.
